# Resection of Non-Functional Pancreatic Neuroendocrine Neoplasms—A Single-Center Retrospective Outcome Analysis

**DOI:** 10.3390/curroncol28040268

**Published:** 2021-08-11

**Authors:** Kirsten Lindner, Daniel Binte, Jens Hoeppner, Ulrich F. Wellner, Dominik M. Schulte, Sebastian M. Schmid, Kim Luley, Inga Buchmann, Lars Tharun, Tobias Keck, Judith Gebauer, Birte Kulemann

**Affiliations:** 1 Department of Surgery, University Medical Center of Schleswig-Holstein-Campus Lübeck, Ratzeburger Allee 160, 23538 Lübeck, Germany; kirsten.lindner@lakumed.de (K.L.); daniel-binte@web.de (D.B.); jens.hoeppner@uksh.de (J.H.); ulrich.wellner@uksh.de (U.F.W.); tobias.keck@uksh.de (T.K.); 2 Division of Endocrinology, Diabetology and Clinical Nutrition, Department of Internal Medicine I, University Medical Center Schleswig-Holstein-Campus Kiel, 23538 Lübeck, Germany; dominik.schulte@uksh.de; 3 Institute for Endocrinology and Diabetes, University of Lübeck, Ratzeburger Allee 160, 23538 Lübeck, Germany; sebastian.schmid@uni-luebeck.de (S.M.S.); judith.gebauer@uksh.de (J.G.); 4 Clinic for Hematology and Oncology, University Hospital Schleswig-Holstein-Campus Lübeck, 23538 Lübeck, Germany; kim.luley@uksh.de; 5 Department of Radiology and Nuclear Medicine, University of Lübeck, Ratzeburger Alle 160, 23538 Lübeck, Germany; inga.buchmann@uksh.de; 6 Institute of Pathology, University Medical Center of Schleswig-Holstein-Campus Lübeck, Ratzeburger Allee 160, 23538 Lübeck, Germany; lars.tharun@uksh.de; 7 Faculty of Medicine, University of Freiburg, Hugstetter Str. 55, 79106 Freiburg, Germany

**Keywords:** pancreatic neuroendocrine neoplasm, postoperative complications, pancreatic fistula, outcome, G2 pNEN

## Abstract

Surgery remains the only curative treatment of pancreatic neuroendocrine neoplasms (pNEN). Here, we report the outcome after surgery for non-functional pNEN at a European Neuroendocrine Tumor Society (ENETS) center in Germany between 2000 and 2019; cases were analyzed for surgical (Clavien–Dindo classification; CDc) and oncological outcomes. Forty-nine patients (tumor grading G1 *n* = 25, G2 *n* = 22, G3 *n* = 2), with a median age of 56 years, were included. Severe complications (CDc ≥ grade 3b) occurred in 11 patients (22.4%) and type B/C pancreatic fistulas (POPFs) occurred in 5 patients (10.2%); in-hospital mortality was 2% (*n* = 1). Six of seven patients with tumor recurrence (14.3%) had G2 tumors in the pancreatic body/tail. The median survival was 5.7 years (68 months; [1–228 months]). Neither the occurrence (*p* = 0.683) nor the severity of complications had an influence on the relapse behavior (*p* = 0.086). This also applied for a POPF (≥B, *p* = 0.609). G2 pNEN patients (*n* = 22) with and without tumor recurrence had similar median tumor sizes (4 cm and 3.9 cm, respectively). Five of the six relapsed G2 patients (83.3%) had tumor-positive lymph nodes (N+); all G2 pNEN patients with recurrence had initially been treated with distal pancreatic resection. Pancreatic resections for pNEN are safe but associated with relevant postoperative morbidity. Future studies are needed to evaluate suitable resection strategies for G2 pNEN.

## 1. Introduction

In pancreatic neuroendocrine neoplasms (pNEN), the association between the surgical access [1,2], the extent of resection [3,4,5], simultaneous lymphadenectomy [6] and the patient’s oncological outcome is a subject of discussion. However, severe postoperative complications are frequent and occur in 15.6% of the patients after surgery for gastro-entero-pancreatic neuroendocrine neoplasm (NEN) [7].

In particular, pancreatic surgery continues to be associated with high morbidity. In pancreatic cancer patients, studies have shown reduced long-term survival in the presence of postoperative complications [8]. Higher tumor recurrence rates are especially discussed in the case of postoperative pancreatic fistula (POPF) [9].

In resected gastro-entero-pancreatic NEN, 27% of all patients suffer from tumor recurrence within three years after surgery [10]. Several studies focused on patient-specific risk stratification in pancreatic surgery [11,12]. In this context, a risk score was defined to plan pragmatic aftercare. However, apart from extensive wound defects and deep vein thrombosis, the prognostic influence of postoperative complications on tumor recurrence was not evaluated in patients with pNEN [11]. Given the option of various resection methods, including parenchyma-sparing surgery and robot-assisted resection up to multivisceral resections in pancreatic NEN, factors for postoperative morbidity and tumor relapse should be considered when planning the surgical management of pNEN patients. In particular, the extent of resection for G2 pNEN is under debate [12,13].

The present study evaluated oncological outcomes after pNEN resection in a single European Neuroendocrine Tumor Society (ENETS) center in Germany. Referring to the discussion about the adequate extent of resection in pancreatic NEN and the known high morbidity rate of pancreatic resections in general, this study focused on the influence of postoperative complications on the occurrence of tumor relapse and long-term survival.

## 2. Materials and Methods

During the study period from January 2000 to December 2019, 87 patients with pNEN were treated at the Department of Surgery, University Medical Center of Schleswig-Holstein, Campus Lübeck. A total of 63 patients underwent pancreatic resection, and 24 patients were unresectable. Of the resected patients, 14 patients had a functionally active pNEN (insulinoma *n* = 11, gastrinoma *n* = 1, glucagonoma *n* = 2). These were excluded from the study because insulinoma, in particular, has a very low recurrence rate and would therefore falsify the result. Forty-nine patients were finally included for further analysis; they underwent a pancreatic resection due to non-functional (NF) pancreatic NEN. The median follow-up was 5.6 years (68 months).

We analyzed demographic data and surgical procedures, including the postoperative course and the occurrence of complications. The 2017 WHO classification was used for the tumor classification. Findings from previous years were revised accordingly. Following this, the differentiation of the tumor grading was classified as follows: NET G1 with a Ki-67 index < 3%, NET G2 3–20%, NET G3 > 20% [14]. Two patients suffered from NET G3, with a Ki-67 index of 25% and 40%. Complications were classified according to the Clavien–Dindo classification (CDc) [15] (Table 1).

Postoperative pancreatic fistula (POPF) was classified according to the International Study Group of Pancreatic Surgery (ISGPS) as types A, B and C—whereby only types B and C describe clinically relevant fistulas [16]. In addition to median overall survival, the occurrence of relapse and relapse-free survival was recorded. Recurrence of disease was defined as evidence of any suspicious lesion found on imaging which suggested a recurrence of disease, using MRI, CT and somatostatin receptor scintigraphy, with or without tissue biopsy histological confirmation. Follow-up for disease recurrence was based on the applicable ENETS guidelines per individual protocol.

Three patients had synchronous liver metastasis at the time of resection. In these cases, the occurrence of further tumor manifestations was defined as tumor recurrence. Progression-free survival (PFS) was calculated as the median of actual survival from the time of surgery, and differences in survival were tested by the logrank test. Two patients were excluded from the PFS analysis due to lacking follow-up data. Multivariate analysis for tumor recurrence was calculated with a logistic regression model using the forward conditional approach.

Results were expressed as median value/range or percentage. Statistical significance of differences between groups was determined using the chi-square test and the *t*-test. A value of *p* < 0.05 was considered statistically significant (SPSS, Chicago, IL, USA).

## 3. Results

From 1 January 2000 to 31 December 2019, 49 patients (median age 56, range 15–86, male 45%) were included who underwent pancreatic resection due to pNEN.

### 3.1. Patients and Characteristics

Most patients had tumors in the pancreatic body/tail (61.2%); the most common resection was a distal pancreatectomy (40.8%). The number of patients was equally distributed among pT1, pT2 and pT3 tumors (32.7%, 26.5% and 34.7%, respectively), and about half of the patients did not show lymph node metastases (pN0 = 51%). Most tumors were either G1 (51%) or G2 (44.9%) tumors, with only two G3 tumors in the cohort. Liver metastases were detected histologically in three patients by taking a sample. There was no liver resection. Demographic and clinicopathological characteristics of the study cohort are summarized in Table 2.

Table 1 shows postoperative complications. One patient died during the hospital stay. He had undergone emergency operation due to Bouveret syndrome with an incidental finding of a pancreatic NEN and suffered from complications during the postoperative course. In general, severe postoperative complications requiring general anesthesia (CDc ≥ grade 3b) occurred in 11 patients (22.4%), while 10 patients needed a postoperative intervention (CDc grade 3a; 20.4%). Pancreas-specific reoperations (CDc 3b) were performed due to postoperative bleeding in three cases, anastomotic leak in two cases and one perforation of the naso-gastric tube in the jejunal loop after multivisceral resection. The other two re-interventions were performed due to cervical infection of the central vein catheter with a subcutaneous abscess and one deep wound infection. A POPF ≥ grade B occurred in five patients (10.2%) (Table 3).

Table 3 presents relevant prognostic factors in terms of tumor recurrence. Well-established prognostic factors for tumor relapse were confirmed on univariate analysis including tumor size, T stage, tumor grading and metastases. The occurrence of postoperative complications had no influence on the relapse behavior, the occurrence of complications in general (*p* = 0.683) or the differentiation of the severity in detail (CDc ≥ grade 3b, *p* = 0.086). This also applied to a POPF (*p* = 0.744). Multivariate logistic regression revealed no independent risk factor for patterns of recurrence. The most frequent site of tumor relapse in our patients was the liver (5/7), followed by bone metastasis (3/7) and intraabdominal lymph nodes (3/7).

The median survival of the overall collective was 5.7 years (68.5 months; range 1–225 months). In seven patients (14.3%), tumor recurrence was demonstrated in the follow-up period: in terms of the median, this was at 24 months (range 5–49) after the resection. The median PFS was 58 months (range 1–225). T stage, M stage and tumor grading had a significant impact on PFS. For example, none of the 16 patients with pT1 pNEN developed recurrence. In contrast, there was no difference in PFS if severe complications or a POPF occurred (see Table 4).

### 3.2. G2 Tumors

In this study, 22 patients had a histopathological G2 tumor (see Table 5). The gender distribution was almost equal. Six of the patients suffered from relapse (27.3%). Interestingly, the tumor size was also almost the same in both groups: 4 cm in the recurrence group, and 3.9 cm in the non-recurrence group. All pNENs of patients with tumor recurrence were located in the pancreatic body or tail, and all patients underwent a distal pancreatic resection, including one multivisceral resection.

Patients without tumor recurrence showed an 81.3% complete R0 resection versus 66.7% of the patients with recurrence. Tumor recurrence was not associated with the observed complications (CDc grade ≥ 3a) or POFPs (Table 5).

## 4. Discussion

In the current study, we analyzed 49 patients with pancreatic resections for non-functional pNEN. Postoperative complications occurred in 77.6%, and severe complications with CDc ≥ 3b occurred in 22.4% of the patients. This is in line with the current literature on pancreatic resection and surgery for neuroendocrine tumors [11,12]. Neither severe postoperative complications nor POPFs, in particular, influenced the occurrence of a tumor relapse.

The prognostic relevance of the established risk factors for tumor recurrence was confirmed, including T stage and the tumor grading, while tumor positivity of lymph nodes did not reach statistical significance for PFS (*p* = 0.51) [17]. No patient with a pT1 tumor or a G1 stage pNEN developed tumor recurrence.

Postoperative complications—particularly the influence of POPFs—are under discussion as they may affect the local and systemic immune response and thus also the occurrence of relapses in pancreatic carcinoma [9,18]. Only limited studies are available to discuss POPFs in pNEN, even though POPFs are reported to occur more often in pNEN than in pancreatic adenocarcinoma. This is most likely due to the softer consistency of the pancreas itself or the resection technique with a normal diameter of the Wirsung duct [11]. In the present study, 10.2% of all patients developed a POPF with a prolonged drain in situ, and the need for an interventional drain placement or reoperation (*n* = 2) (ISGPS grades B–C); there was no connection between the postoperative occurrence of a POPF and tumor recurrence.

One other study also focused on the impact of postoperative complications after resection of pNEN [19]. Interestingly, the rate of tumor recurrence was higher than in our study (28.5% versus 14.3%). Additionally, the median time to tumor recurrence was distinctly shorter than in the present study (11.7 months versus 24 months). However, as in our observation, no connection between postoperative morbidity and tumor relapse was demonstrated.

Seven patients in our study (14.3%) developed tumor recurrence. This is in line with reported recurrence rates of 13–36% [20,21,22]. Additionally, the median time of recurrence of 24 months was comparable to other studies that reported 6 to 38 months [20]. T stage and the tumor grading significantly influenced the progression-free survival. The most frequent site of tumor relapse was the liver (5/7), followed by bone metastasis (3/7) and intraabdominal lymph nodes (3/7). Recent registry analysis from the SEER database on pNEN patients who underwent pancreatic resection also described the liver as the main site of tumor relapse [23].

In a subgroup analysis, the relapse rate in the 22 patients with pNEN G2 was evaluated. Of these, six patients (27.3%) had a tumor relapse. The median tumor size was 4 cm. All patients with relapse had a prior distal pancreatectomy, which in 66.7% of the cases was an R0 resection. The occurrence of postoperative complications, including a POPF, did not affect the rate of recurrence.

The group of “well-differentiated” G1 and G2 pNENs is prognostically more advantageous than G3 tumors in the literature; in particular, G1 tumors have an excellent prognosis [22]. The differentiation between pNEN G1 and G2 after curative resection was already shown to be prognostically crucial for the occurrence of relapse in other studies [19,24]. This was confirmed in our cohort: no patient with a G1 tumor (*n* = 25) developed a tumor relapse.

Another decisive factor for the prognosis of pNEN patients is a complete tumor (R0) resection [5,25,26]. In the context of relevant postoperative morbidity in pancreatic surgery, the “right” extent of surgery for each patient is under debate—and often discussed in interdisciplinary tumor conferences. The option of a limited resection seems to be reserved for patients with a small pNEN. This applies primarily to G1 pNEN, but also to G2 pNEN; here, the adequate preoperative staging, including the exclusion of lymphoid metastasis, is of great importance [27,28,29].

The present study has several limitations: Its retrospective design with all known disadvantages allows only limited conclusions. Moreover, the collective size of this rare tumor entity is small. The effects of additional adjuvant therapy, especially after R1 resection, are uncertain. Besides that, pNENs are very heterogeneous, which is also reflected in the study collective. This refers not only to the tumor characteristics but also to the surgical procedures evaluated.

The question of whether patients with locally advanced pNEN G2 benefit from extensive resection, including venous resection and multivisceral resection, with a possibly higher complication rate, is still under discussion [30,31,32,33]. Our findings support the possibilities for a more aggressive resection approach in G2/N+ tumors based on the three following findings.

First, patients with tumor recurrence had undergone “only” distal pancreatic resection, while the majority of patients in the non-recurrence group had undergone more extensive surgery. Second, postoperative complications were not associated with an adverse oncological outcome. Third, only 2/3 of the patients with tumor recurrence underwent complete resection (66.7% R0 resection). Multicentric trials with higher patient numbers are needed to finally confirm these findings.

## 5. Conclusions

Against the background of the high complication rate in pancreatic surgery and the relevant incidence of POPFs, the present study showed no connection between postoperative morbidity and tumor recurrence or recurrence-free survival. Given the complexity of sophisticated diagnostics and therapy planning, interdisciplinary tumor conferences and patient care in experienced centers are crucial for pNEN patients.

## Figures and Tables

**Table 1:** Postoperative complications classified according to Clavien–Dindo (CDc) and the International Study Group of Pancreatic Surgery (ISGPS).

Variable	Definition	Number (*n* = 49)	%
Clavien–Dindo			
Grade 0	No complication	11	22.4
Grade 1	Antiemetic treatment/drug treatment	5	10.2
Grade 2	For example, blood transfusion/no intervention	12	24.5
Grade 3a	Intervention without general anesthesia	10	20.4
Grade 3b	Intervention in general anesthesia	6	12.2
Grade 4a	ICU, one organ failure	2	4.1
Grade 4b	ICU, multiorgan failure	2	4.1
Grade 5	death	1	2.0
ISGPS classification			
No postoperative pancreatic fistula		29	59.2
Grade A	Biochemical leak, no clinical relevance	15	30.6
Grade B	Drain > 3 weeks, interventional drainage, angiographic intervention	3	6.1
Grade C	Organ failure, reoperation, death	2	4.1

**Table 2:** Demographic and clinicopathological characteristics of the overall collective.

Variable	Number (*n* = 49)	%
Age (Median)	56 years (range 15–86)	
Sex (Male)	25	51
Tumor localization		
Pancreatic head	19	38.8
Pancreatic body/tail	30	61.2
Tumor size (Median)	2.8 cm (range 0.4–15)	
Surgical procedure		
Pancreaticoduodenectomy	15	29.6
Distal pancreatectomy	20	40.8
Central pancreatic resection	2	4.1
Total pancreatectomy	5	10.2
Enucleation	7	14.3
Minimally invasive (yes) *	25	51
Robot-assisted	8	16.3
Conversion	2	4.1
Portal vein resection	4	8.2
Mutivisceral resection	5	10.2
Tumor stage		
pT1 (<2 cm)	16	32.7
pT2 (2–4 cm)	13	26.5
pT3 (>4 cm pancreas/duodenum)	17	34.7
pT4 (outside pancreas/duodenum)	3	6.1
N stage		
pN0	25	51
pN1	18	36.7
pNx	6	12.2
M stage		
pM0	46	93.9
pM1	3	6.1
R0 resection (yes)	43	87.8
Grading		
G1	25	51
G2	22	44.9
G3	2	4.1

* Twenty-four patients were resected in an open fashion.

**Table 3:** Factors influencing the risk for tumor relapse.

Parameter	No Tumor Recurrence (*n* = 42) (%)	Recurrence (*n* = 7) (%)	*p*-Value Univariate	*p*-Value Multivariate
Male	21 (50)	4 (57.1)	0.524	
Age *	57 (29–86)	52 (15–77)	0.318	
Tumor size (cm) *	2.3 (0.4–15)	4.0 (3–9)	**0.049**	0.131
Tumor localization			0.161	
Pancreatic head	18 (42.9)	1 (14.3)		
Pancreatic body/tail	24 (47.1)	6 (85.7)		
Laparoscopic resection	22 (52.4)	3 (42.9)	0.476	
R0 resection	38 (90.5)	5 (71.4)	0.199	
Portal vein resection	4 (9.5)	0	0.528	
Multivisceral resection	3 (7.1)	2 (28.6)	0.143	
Grading			**0.010**	0.202
G1	25 (59.9)	0		
G2	16 (38.1)	6 (85.7)		
G3	1 (2.4)	1 (14.3)		
T stage			**0.033**	0.322
pT1	16 (38.1)	0		
pT2	12 (28.6)	1 (14.3)		
pT3	12 (28.6)	5 (71.4)		
pT4	2 (4.8)	1 (14.3)		
N stage			0.088	0.983
N0	24 (57.1)	1 (14.3)		
N1	13 (31)	5 (71.4)		
Nx	5 (11.9)	1 (14.3)		
M stage			**0.050**	0.987
M0	41 (97.6)	5 (71.4)		
M1	1 (2.4)	2 (28.6)		
Clavien–Dindo grade ≥ 3a	21 (50)	1 (14.3)	0.086	0.082
ISGPS grade B + C	5 (10.2) **	0	0.744	0.774

Significant values are printed in bold. ISGPS = International Study Group of Pancreatic Surgery. * median, range; ** 5 patients developed a pancreatic fistula grade B or C in the overall collective.

**Table 4:** Median progression-free survival and univariate Cox regression analysis.

Parameter		Number (*n* = 47 *)	Tumor Recurrence (*n* = 7)	Median PFS	HR	95% CI		*p*-Value
						Lower	Upper	
Sex	Female	24	3	57.5 (5–191)	1.283	0.287	5.746	0.744
	Male	23	4	68 (1–215)				
Age	≤68	40	6	63.5 (1–215)	1.079	0.129	9.023	0.944
	>68	7	1	32 8(71–86)				
Tumor size	≤2 cm	15	0	71 (8–176)	40.564	0.62	64.972	0.257
	>2 cm	32	7	46 (1–215)				
Resection	PPPD	15	1	36 (5–191)	0.805	0.483	1.341	0.405
	Distal	19	6	70 (5–215)				
	Central	2	0	54.5 (41–68)				
	Total	4	0	37.5 (1–70)				
	Enucleation	7	0	85 (8–170)				
R status	R0	42	5	58.8 (1–215)	2.214	0.428	11.449	0.343
	R1	5	2	57 (25–70)				
T stage	T1	15	0	72 (8–176)	3.295	1.300	8.354	**0.012**
	T2	13	1	59 (5–215)				
	T3	16	5	35.5 (1–77)				
	T4	3	1	20 (11–128)				
N stage	N0	24	1	70 (6–191)	1.077	0.863	1.344	0.511
	N1	17	5	49 (1–215)				
	Nx	6	1	40.5 (8–85)				
M stage	M0	45	5	59 (1–215)	8.780	1.692	45.555	**0.016**
	M1	2	2	36.5 (24–49)				
G stage	G1	25	0	72 (8–215)	17.7	3.144	99.651	**0.001**
	G2	15	6	41 (1–218)				
	G3	0	1	11				
Clavien–Dindo	0–2	22	6	36 (5–170)	5.888	0.704	49.234	0.102
	≥3a	20	1	69.5 (1–215)				
ISGPS	0	28	4	69.5 (1–215)	0.793	0.292	2.151	0.649
	A	14	3	26.5 (7–176)				
	B	3	0	85 (77–109)				
	C	2	0	68				

* Two patients were excluded as PFS was unknown. Significant values are printed in bold. PPPD = pancreaticoduodenectomy, Distal = distal pancreatectomy, Central = central pancreatic resection, Total = total pancreatectomy, PFS = progression-free survival.

**Table 5:** Comparison of the patients with and without tumor recurrence in G2 pNEN (*n* = 22).

Parameter	No Tumor Recurrence (*n* = 16) (%)	Recurrence (*n* = 6) (%)	*p*-Value
Male	9 (56.3)	4 (66.7)	0.523
Tumor size (cm) *	3.9 (0.7–12.5)	4.0 (3–9)	0.267
Tumor localization			0.076
Pancreatic head	7 (43.8)	0	
Pancreatic body/tail	9	6 (100)	
Surgical procedure			0.078
Pancreaticoduodenectomy	4 (25)	0	
Distal pancreatectomy	4 (25)	6 (100)	
Central pancreatic resection	1 (6.3)	0	
Total pancreatectomy	5 (31.3)	0	
Enucleation	2 (12.5)	0	
Laparoscopic resection	8 (50)	3 (50)	0.682
R0 resection	13 (81.3)	4 (66.7)	0.419
Portal vein resection	3 (18.8)	0	0.364
Multivisceral resection	2 (12.5)	1 (16.7)	0.636
T stage			0.353
pT1	3 (18.8)	0	
pT2	4 (25)	1 (16.7)	
pT3	7 (43.8)	5 (83.3)	
pT4	2 (12.5)	0	
N stage			0.339
N0	6 (37.5)	1 (16.7)	
N1	8 (50)	5 (83.3)	
Nx	2 (12.5)	0	
M stage			0.065
M0	16 (100)	4 (66.7)	
M1	0	2 (33.3)	
Clavien–Dindo grade ≥ 3a	7 (43.8)	1 (16.7)	0.255
ISGPS grade B + C	1 (6.3)	0	0.662

* Median, range. ISGPS = International Study Group of Pancreatic Surgery.

## Data Availability

The data presented in this study are available on request from the corresponding author. The data are not publicly available due to patient privacy.
